# Cellular Interactions of Cardiac Repair After Myocardial Infarction

**DOI:** 10.3390/cells14231903

**Published:** 2025-12-01

**Authors:** Merry L. Lindsey, Ashton F. Oliver, Amadou Gaye, Pius N. Nde, Kristine Y. DeLeon-Pennell, Germán E. González

**Affiliations:** 1 Department of Biomedical Sciences, Meharry Medical College, Nashville, TN 37208, USA; ashton.oliver@mmc.edu (A.F.O.); pnde@mmc.edu (P.N.N.); 2 Department of Applied Education, Meharry Medical College, Nashville, TN 37208, USA; 3 Research Service, Nashville VA Medical Center, Nashville, TN 37212, USA; 4 Department of Integrative Genomics and Epidemiology, Meharry Medical College, Nashville, TN 37208, USA; amadou.gaye@mmc.edu; 5 Division of Cardiology, Department of Medicine, School of Medicine, Medical University of South Carolina, Charleston, SC 29425, USA; deleonky@musc.edu; 6 Research Service, Ralph H. Johnson Veterans Affairs Medical Center, Charleston, SC 29401, USA; 7 Laboratorio de Patología Cardiovascular Experimental e Hipertensión Arterial, Instituto de Investigaciones Biomédicas (UCA-CONICET), Facultad de Ciencias Médicas, Universidad Católica Argentina, Buenos Aires C1107, Argentina; 8 Departamento de Patología, Instituto de Salud Comunitaria, Universidad Nacional de Hurlingham, Villa Tesei B1688, Buenos Aires, Argentina

**Keywords:** cardiac wound healing, inflammation, leukocytes, macrophage, neutrophil, lymphocyte, fibroblast, scar formation

## Abstract

**Highlights:**

**What are the main findings?**

- Myocardial infarction (MI) triggers a cascade of cellular and molecular events, including rapid infiltration of inflammatory cells to remove necrotic tissue.
- Various cell populations, particularly leukocytes and fibroblasts, play crucial roles in tissue remodeling and scar formation.

**What are the implications of the main findings?**

- While scar formation is vital for structural stabilization, it also leads to increased myocardial stiffness and impaired contractile function.
- Understanding the cellular dynamics and inflammatory signaling in MI healing can inform therapeutic strategies to enhance recovery outcomes.

**Abstract:**

When blood flow to a part of the myocardial muscle is reduced or blocked, it leads to tissue ischemia in that region. Myocardial infarction (MI) occurs when the ischemic insult is of sufficient duration in time to induce cardiomyocyte death and subsequent activation of the innate immune response. MI initiates a complex cascade of cellular and molecular events within the left ventricle. Inflammatory cells rapidly infiltrate the infarcted area to remove necrotic tissue, setting the stage for reparative wound healing processes. Over the ensuing days, various cell populations—including leukocytes, fibroblasts, and endothelial cells—are attracted to the infarcted site by inflammatory cytokines and chemokines. The activated cells at the site of injury contribute to tissue remodeling and scar formation through the deposition of extracellular matrix components, particularly collagen. While scar formation is essential for structural stabilization of the infarct region to replace the loss of cardiomyocytes, scar tissue also increases myocardial stiffness and impairs cardiac contractile function. This review summarizes our knowledge regarding cellular dynamics, inflammatory signaling, and cardiac remodeling that govern MI healing. We identify the current gaps in the field and provide a foundational resource for those seeking to understand the biological underpinnings of cardiac repair following MI.

## 1. Introduction

Myocardial infarction (MI) occurs when myocardial ischemia is of sufficient duration to induce cardiomyocyte death. The ischemic insult initiates a complex and highly orchestrated wound-healing response in the left ventricle downstream of the reduced blood flow. Cardiac wound healing progresses through distinct yet overlapping phases: inflammation, anti-inflammation and resolution, and tissue repair culminating in scar formation [1,2,3]. The inability of cardiomyocytes to regenerate necessitates the replacement of necrotic tissue with an extracellular matrix (ECM)-rich scar, a process that is essential for maintaining structural integrity and preventing complications such as ventricular rupture [4,5]. Understanding the cellular and molecular mechanisms that oversee this response is important for developing therapeutic strategies to mitigate adverse remodeling and reducing the risk of progression from MI to heart failure. This review focuses on the response to MI in the mouse model of permanent coronary artery occlusion [6,7].

This review summarizes our current knowledge on the mechanisms of cardiac remodeling after MI, focusing on five major themes: the inflammatory response, ECM dynamics, cellular interactions, therapeutic implications, and knowledge gaps. Our goal is to provide trainees and early-career researchers with a foundational understanding of the temporal and spatial coordination of cellular events that occur during infarct healing. By elucidating the roles of critical cell types and signaling pathways, this review can help inform future research directions.

## 2. Initiation of the MI Inflammatory Response

The timeline for the cardiac response to MI is summarized in Table 1 [3,8,9,10]. MI triggers the release of damage-associated molecular patterns (DAMPs) such as High-Mobility Group Box 1 (HMGB1), S100A8/A9, and mitochondrial DNA from necrotic cardiomyocytes, which activate pattern recognition receptors on resident immune cells and fibroblasts [1,11,12]. This leads to the secretion of pro-inflammatory cytokines and chemokines, complement activation, and increased vascular permeability, facilitating leukocyte infiltration [13,14,15].

The inflammatory phase begins within hours of MI and is marked by infiltration of leukocytes (lymphocytes, monocytes/macrophages, and neutrophils) into the infarcted myocardium [10,16,17]. Leukocytes release proteases including matrix metalloproteinases (MMP)-7, MMP-8 and MMP-9, which degrade the existing ECM to facilitate the clearance of necrotic debris [18,19,20]. Leukocytes also secrete pro-inflammatory cytokines including interleukin (IL)-1β, tumor necrosis factor alpha (TNF-α), and CXCL chemokines to amplify the inflammatory response and recruit additional leukocytes [21,22].

**Table 1:** Timeline of Cell Responses to MI [1,2,11,16,18,23,24,25,26,27].

MI Days	Cell	Key Activities	Markers/Signals
0–1	Cardiomyocytes	Necrosis	DAMPs
	Macrophages, Lymphocytes, Neutrophils	Infiltration, Pro-inflammation cytokine & chemokine release, ECM degradation & debris clearance	Cxcl1/2/8, IL-1β, MMP-8, MMP-9
1–3	Macrophages, Lymphocytes, Neutrophils	Peak infiltration, Pro-inflammation	Il-6, Tnf-α, Tgf-β, Csf-1
	Fibroblasts	Amplified breakdown of ECM	
3–5	Macrophages, Lymphocytes, Neutrophils	Transition to anti-inflammatory phenotype, Resolution of inflammation, Phagocytosis of apoptotic cells	Arg1, IL-10, Lgals3, Tgf-β, Vegf
	Fibroblasts (proliferative),	ECM deposition, Stimulation of Angiogenesis	Fn, Lgals1, Lgals3, Smad
	Endothelial cells (angiogenesis)	Revascularization of the infarct region	Smad, Vegf
5–7	Fibroblasts (scar-forming)	Scar formation, ECM maturation, anti-Angiogenesis	Col I, Col III, Sparc, Lgals1, Lgals3, Lox, TIMPs
	Lymphocytes, Macrophages, Neutrophils	Reparative phenotype, anti-angiogenic Signaling	Tsp-1
	Endothelial cells	Anti-angiogenic signaling	TIMPs
7+	Fibroblasts, Macrophages	Scar maturation, Suppression of remaining inflammation, Maintenance of new homeostasis; Stabilization of neovasculature	Crosslinked ECM (collagens), Tsp-1

Inflammation is necessary for initiating repair, as inhibiting the inflammatory response with carprofen treatment prevents the acute inflammatory response and yields an extended period of non-resolving inflammation after MI [28]. While an acute inflammatory response is necessary for initiating repair mechanisms, excessive or sustained inflammation can become maladaptive. In this context, chronic inflammatory signaling may exacerbate tissue injury, disrupt cellular homeostasis, and interfere with the coordinated progression of healing [29,30]. Therefore, timely resolution of inflammation is critical, and this transition is mediated by neutrophil apoptosis and macrophage polarization toward an anti-inflammatory phenotype [31,32,33].

Neutrophils are first responders to MI. Neutrophils infiltrate the infarcted myocardium within hours, peaking around day 1 after MI [11,18,34,35]. Neutrophils release proteolytic enzymes including MMP-8, MMP-9, neutrophil elastase, and myeloperoxidase, which contribute to ECM degradation and debris clearance [18,36,37]. Myeloperoxidase generates reactive oxidant species that directly modify ECM proteins (collagens, elastin, fibronectin) by oxidation, as well as activate proteases such as MMPs to facilitate tissue breakdown and clearance of necrotic tissue [38,39]. Neutrophils also produce reactive oxygen species and neutrophil extracellular traps that contribute to sterile inflammation [40,41,42]. Sterile inflammation is a type of inflammatory response that occurs without the presence of an infectious agent; in this case, it is triggered by tissue injury. There is a dual role of neutrophils in the response to MI [34]. Neutrophils are essential for initiating repair and at the same time can exacerbate injury if overactivated. Excessive ECM degradation by neutrophils increases the risk of infarct wall thinning and rupture [21,43].

Inflammation peaks between days 1 and 3 after MI, and timely resolution is needed to prevent adverse remodeling [3,17,44]. Adverse cardiac remodeling refers to the detrimental alterations in myocardial structure, shape, and physiology following MI [45,46]. These maladaptive changes can lead to the development of heart failure. Adverse cardiac remodeling encompasses various molecular, cellular, and ECM changes within both the remote and infarct regions [46,47].

Resolution of inflammation is mediated by lipoxin A4 and resolvin D1 [48]. Resolution mediators promote the healing process by facilitating clearance of inflammatory cells and promoting tissue repair to ultimately restore homeostasis [32,33,49,50]. While there have been a number of strategies applied to inhibit the MI inflammation response, few approaches have focused on promoting the resolution of inflammation to improve outcomes [32].

As inflammation subsides within the left ventricle, the reparative phase begins and is denoted by conversion of leukocytes to an anti-inflammatory phenotype and activation of fibroblasts and endothelial cells to promote synthesis of new vessels and ECM [51]. For decades, we have understood that fibroblasts proliferate and differentiate into a state that has traditionally been referred to as a myofibroblast. Myofibroblasts are characterized by the expression of α smooth muscle actin and are stimulated by transforming growth factor-beta (TGF-β) to produce collagen I and III, fibronectin, and other ECM components that form the structural basis of the infarct scar [16,52]. We now know that fibroblast activation is more complex than originally thought, with fibroblasts transitioning from pro-inflammatory cells at day 1 MI to anti-inflammatory cells that promote activation of endothelial cells at day 3 MI to scar-forming cells by day 7 MI [23,53]. Concurrently, angiogenesis is stimulated by vascular endothelial growth factor (VEGF) and fibroblast growth factor (FGF) to promote revascularization of the infarct zone [54]. The balance between ECM deposition and degradation during this phase determines the net quality and stability of the scar, with implications for long-term cardiac physiology [10].

The maturation phase of the response to MI involves ECM crosslinking and remodeling, processes that enhance scar tensile strength and prevent adverse ventricular dilation [55]. Enzymes such as lysyl oxidase (LOX) and matricellular proteins like secreted protein acidic and rich in cysteines (SPARC) and thrombospondin-1 (TSP-1) play key roles in this phase [56,57,58]. Importantly, fibroblasts remain in the infarct region throughout the lifespan, and their prolonged presence may contribute to pathological remodeling if not properly regulated [55,59,60]. The dynamic interplay among neutrophils, macrophages, fibroblasts, and endothelial cells underscores the complexity of infarct healing and highlights the need for targeted interventions that modulate specific cellular functions at appropriate time points.

Neutrophil depletion impairs macrophage polarization and phagocytosis, worsening outcomes [31,61]. Circadian rhythms also have an influence on neutrophil physiology, as neutrophils from mice given MI in the evening are more pro-inflammatory than neutrophils from mice given MI in the morning [62]. Circadian oscillations of neutrophil recruitment into the MI region determine infarct size, healing, and cardiac physiology. MI neutrophils transition from having pro-inflammatory to anti-inflammatory phenotypes by day 3 MI. At this time, existing neutrophils within the infarct region undergo apoptosis and are phagocytosed by macrophage efferocytosis [63,64]. Efferocytosis of apoptotic neutrophils is a key driver of macrophage repolarization to M2-like, pro-reparative phenotypes, which in turn shapes the reparative microenvironment (increased IL-10/TGF-β) that favors resolution and activation of matrix-restorative processes [37,64,65,66]. Newly infiltrating neutrophils express an anti-inflammatory N2 phenotype [11,21,43,67]. Neutrophils begin expressing reparative markers including galectin (Lgals)-3, fibronectin, and tissue inhibitor of metalloproteinases (TIMP)-2, contributing to ECM synthesis and scar formation [18].

Monocytes are recruited in response to chemokines like monocyte chemotactic protein-1 (Mcp-1, Ccl2) and differentiate into macrophages [67]. Initially, macrophages adopt a pro-inflammatory phenotype, secreting Tnf-α, Il-1β, and MMPs to degrade ECM and clear necrotic debris [43]. Over the first seven days of MI, macrophages polarize, transitioning from pro-inflammatory (M1) to anti-inflammatory and reparative (M2) phenotypes [68]. IL-10 supplementation starting at day 1 MI improves repair by stimulating M2 macrophage polarization and fibroblast activation [69].

The temporal sequence of immune activation after MI seen in experimental mouse models of MI closely parallels clinical observations, although reperfusion profoundly accelerates and intensifies these events. In humans, the molecular mediators that link the different phases of healing—IL-1β, TNF-α and IL-6 in the early inflammatory window, and TGF-β, MMPs, and TIMPs during the reparative phase—are detectable systemically in patients with MI and correlate with adverse electrophysiological remodeling, infarct expansion, and later heart-failure phenotypes [29]. Timely reperfusion after MI also triggers inflammation, modifies the balance between necessary debris clearance and maladaptive matrix degradation (through enhanced myeloperoxidase activity, MMP activation and TIMP inactivation), which helps explain why reperfused infarcts often show smaller transmural scar but a distinct pattern of border-zone injury and different arrhythmic risk compared with non-reperfused infarcts [70].

This shift in polarization phenotypes is an important contributor to resolution of inflammation. By day 3 MI, macrophages transition to an anti-inflammatory (M2) phenotype, stimulated by phagocytosis of apoptotic neutrophils and exposure to IL-10 and Tgf-β [69]. Anti-inflammatory macrophages secrete Vegf and Pdgf, promoting angiogenesis and fibroblast activation [23]. Excessive or prolonged pro-inflammatory macrophage presence is linked to adverse remodeling and increased risk of cardiac rupture [71].

Cardiac fibroblasts adopt a pro-inflammatory phenotype during the first days after MI, releasing cytokines and chemokines including Il-1β that recruit leukocytes [16,23]. Day 1 MI fibroblasts also express colony-stimulating factor (CSF)1 to support macrophage differentiation [24]. Between days 3 and 7 after MI, neutrophils decline, while macrophages shift toward an anti-inflammatory, reparative phenotype, secreting IL-10, TGF-β, and VEGF to resolve inflammation and stimulate fibroblast and endothelial activation [23]. These events drive the formation of granulation tissue, a hallmark of this stage, characterized by proliferating fibroblasts, angiogenesis, collagen synthesis and extracellular-matrix deposition (mainly type III collagen and fibronectin) [1,72]. Granulation tissue replaces necrotic myocardium and provides a structural scaffold for scar formation. From 1 to 3 weeks after MI, myofibroblast differentiation, angiogenesis, and matrix crosslinking dominate, marking the onset of the fibrotic phase [9]. The final formation of fibrotic scar should provide and maintain the structural integrity of the infarct wall [9,73].

In general, lymphocytes are categorically detrimental and inevitably lead to excessive myocardial damage [36,74,75,76,77]. The two main types of lymphocytes, T- and B-cells, make up the central cellular components of adaptive immunity and can be further classified into subsets based on their function and protein expression patterns (e.g., CD4 vs. CD8 T cells). Both CD4+ and CD8+ T-cells have been shown to regulate the infiltration of proinflammatory monocytes [74,78,79]. This may be in part due to cardiac-infiltrating T cells suppressing lymph angiogenesis after MI, through interferon-γ signaling [80]. While few studies have demonstrated a direct mechanistic link, B cells have been linked to inflammatory mediators of fibroblast activation and likely promote fibrosis through increased ECM turnover and deposition [81,82]. More recently, studies have pointed to production of cardiac autoantibodies produced by autoreactive B cells involved in the progression of adverse cardiac remodeling in post-MI [83,84,85]. Targeting B cells as a potential therapeutic strategy to attenuate autoantibody production following MI may be a viable option. Platelets also release chemokines that recruit leukocytes and influence inflammation and remodeling [86,87]. Combined, the day 1 MI environment provides a pro-inflammatory milieu that potentiates necrotic myocyte debridement.

## 3. Extracellular Matrix Dynamics

The ECM provides structural support to the myocardium and plays a dynamic role in cardiac remodeling [88]. Following MI, the ECM undergoes extensive degradation in the early time points and converts to scar formation and reorganization during the later phases. MMPs, particularly MMP-2, MM-7, MMP-8, MMP-9, MMP-12, MMP-14, and MMP-28, are upregulated in the infarcted myocardium and contribute to ECM breakdown [19,20,89,90,91]. The major sources of MMPs in the infarcted left ventricle are leukocytes, primarily neutrophils, macrophages, and lymphocytes. This degradation facilitates immune cell infiltration and removal of necrotic myocardial tissue. Unchecked ECM degradation or excessive ECM deposition are both linked to deleterious outcomes. While ECM degradation is a necessary component of the wound healing process, excessive ECM degradation can weaken the ventricular wall and increase the risk of rupture [16]. At the same time, too much ECM deposition can lead to pathological stiffening and arrhythmia [29,92,93,94]

Fibroblasts are activated in response to inflammatory signals and differentiate into myofibroblasts [24,36,58,76]. These cells are the primary source of ECM proteins such as collagen I and III. Myofibroblasts also express contractile proteins like α-smooth muscle actin, contributing to wound contraction. As the reparative phase progresses, ECM synthesis predominates [95]. Collagen deposition stabilizes the infarcted area and prevents ventricular dilation. Cross-linking enzymes such as LOX enhance the tensile strength of the scar. The balance between ECM degradation and synthesis determines the quality of the scar and the extent of ventricular remodeling. Therapeutic strategies that modulate MMP activity and fibroblast function may improve structural outcomes following MI. Fibroblast-secreted collagen, fibronectin, and matricellular proteins mature the scar and determine its mechanical properties [58].

## 4. Cellular Interactions

Cardiac remodeling is orchestrated by complex interactions among various cell types, including neutrophils, macrophages, fibroblasts, and endothelial cells. These interactions are mediated by cytokines, chemokines, and direct cell–cell contact [96,97]. We discuss here the communication between different cell pairings, with key interactions summarized in Table 2.

Neutrophil–Macrophage Interactions. Neutrophil–macrophage crosstalk is essential for the transition from inflammation to repair. Apoptotic neutrophils are phagocytosed by macrophages, which then adopt a reparative phenotype. This process is critical for resolving inflammation and initiating tissue repair. Neutrophils infiltrate the infarct site within hours, peaking at day 1 MI, releasing proteases (MMP-8, MMP-9), cytokines (IL-1β, Tnf-α), and generating reactive oxygen species that initiate ECM breakdown [18].

Apoptotic neutrophils at day 3 are phagocytosed by infiltrating macrophages; this interaction shifts macrophage phenotype from pro-inflammatory (M1-like) to reparative (M2-like), critical for inflammation resolution and repair initiation [31,98].

**Table 2:** Summary of Key Cellular Interactions.

Interaction	Signaling Mechanisms	Outcome
Neutrophil–Macrophage [13,31,43,99]	Apoptosis signals; IL-10; MMP-12; JAK/STAT	Macrophage polarization to reparative phenotype; Apoptotic neutrophil phagocytosis
Macrophage–Fibroblast [67,100,101]	Tgf-β and Pdgf	Fibroblast activation and ECM production
Macrophage–Endothelial Cell [102,103,104]	Vegf, latent Tgf-β (via MT1-MMP), MMP-2	Angiogenesis in the infarct zone; promote endothelial-to-mesenchymal transition and matrix remodeling that link angiogenesis to scar formation
Endothelial–Fibroblast [16,23,105]	Vegf, Fgf2, Tgf-β, Tsp-1	Angiogenesis and tissue remodeling
Lymphocyte–Macrophage [36]	Ifn-γ, IL-13	Cytokine modulation of resolution & Remodeling
Lymphocyte–Fibroblast [36,74,75,76]	Ccl11, IL-4, Tgf-β, MMP-2, MMP-3, MMP-9	Downregulate macrophage recruitment; ECM production and turnover

Macrophage-derived Mmp-12 contributes to neutrophil apoptosis, further promoting transition to anti-inflammation [90,106].

Macrophage–Fibroblast Interactions. Macrophages influence fibroblast activation through the secretion of Tgf-β and other growth factors. These signals promote fibroblast proliferation, differentiation, and ECM production. Conversely, fibroblasts can modulate macrophage function by secreting cytokines and presenting antigens. Reparative macrophages secrete Tgf-β and Pdgf, which stimulate fibroblast transdifferentiation into myofibroblasts, promoting ECM synthesis and scar formation [100,107]. Fibroblasts, depending on local cytokine milieu, polarize from pro-inflammatory (initially) toward a proliferative, then reparative, and ultimately quiescent phenotype; at day 3, fibroblasts show pro-angiogenic gene expression and ECM synthesis [16,23].

Endothelial Cell–Leukocyte and Fibroblast Interactions. Endothelial cells contribute to neovascularization, which is vital for supplying oxygen and nutrients to the healing myocardium. Endothelial cells interact with both immune cells and fibroblasts to coordinate angiogenesis and tissue remodeling. Activated fibroblasts and macrophages secrete VEGF and other growth factors, stimulating endothelial cell proliferation and neovascularization between days 3–7 [2,108]. Thrombospondin (Tsp)-1 has anti-angiogenic properties, and Tsp-1 secretion by day 7 MI fibroblasts attenuates new vessel formation, contributing to maturation of the scar [16,23].

Disruptions in cellular interactions can impair healing and lead to adverse remodeling. A deeper understanding of intercellular communication networks may reveal novel therapeutic targets to enhance cardiac repair.

## 5. Therapeutic Implications

Current therapies for MI focus on reperfusion and pharmacological management to reduce infarct size and prevent heart failure. These include antiplatelet agents, beta-blockers, ACE inhibitors, and statins [22]. There are several areas of therapeutic promise to limit adverse remodeling after MI. Targeting neutrophil and macrophage polarization, enhancing resolution signals, and modulating fibroblast activation are several avenues that may improve healing and reduce heart failure risk [91,109,110].

Targeting the inflammatory response has been consistently examined over the past 50 years without significant success. Agents that reduce immune cell recruitment, cytokine production, and macrophage polarization, while promising in theory, also have the potential to interfere with necessary cardiac wound healing responses. The one area that has been successfully translated to clinic is the use of IL-1β inhibitors such as canakinumab, which have shown potential in reducing inflammation and improving MI outcomes [111].

Modulation of ECM remodeling is another therapeutic avenue. MMP inhibitors and agents that enhance ECM synthesis or cross-linking may stabilize the infarct and prevent ventricular dilation. A personalized medicine approach that considers individual patient profiles, including genetic and environmental factors, may optimize therapeutic efficacy and minimize adverse effects.

## 6. Knowledge Gaps and Future Directions

Despite advances over the past 30 years that have improved our understanding of MI-driven cardiac remodeling, several knowledge gaps remain (Table 3). One critical area is the long-term impact of acute inflammatory responses on cardiac function and structure. Sex differences in immune responses and remodeling outcomes are not fully understood [112,113]. Research into how hormonal and genetic factors influence these processes may lead to sex-specific therapies. The role of non-coding RNAs, epigenetic modifications, and metabolic reprogramming in cardiac remodeling is an emerging field [114,115,116,117]. How these factors regulate gene expression and cellular behavior during healing remains to be fully understood.

There is a need for better animal models that accurately reflect human MI and remodeling. This includes models that incorporate comorbidities such as diabetes and hypertension. Advancements in single-cell technologies and systems biology approaches to triangulate the interconnections between gene modulation and protein expression may provide a more comprehensive understanding of the cellular and molecular landscape of cardiac remodeling [122,123]. These tools can identify novel targets and biomarkers for therapeutic intervention.

## 7. Conclusions

**Figure 1:**
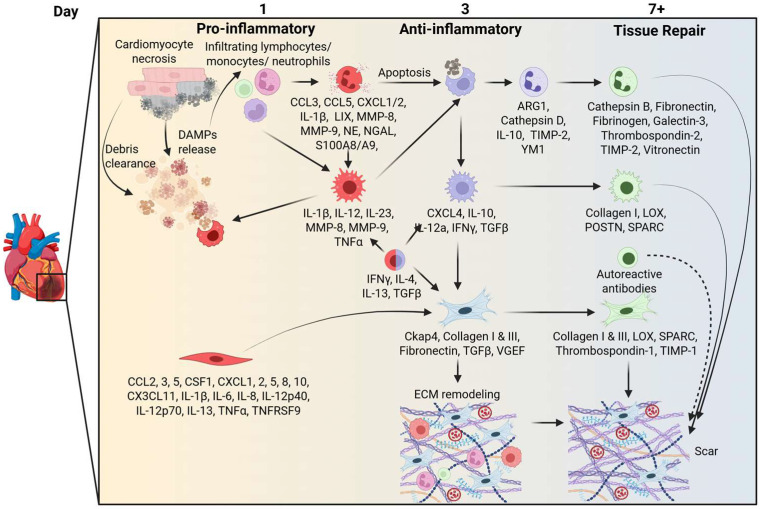
Time course of responses to myocardial infarction. The diagram delineates the temporal cascade of events that occur in response to myocardial infarction (MI), beginning with cardiomyocyte necrosis and damage-associated molecular pattern (DAMP)-mediated activation of the immune response. The pro-inflammatory phase at MI day 1 is characterized by infiltration of lymphocytes, monocytes/macrophages, and neutrophils, along with secretion of cytokines (e.g., interleukin (IL)-1β, tumor necrosis factor (Tnf)-α) and matrix metalloproteinases (MMPs). By day 3, anti-inflammatory mediators (e.g., IL-10 and transforming growth factor (Tgf)-β) and reparative macrophages promote apoptosis and extracellular matrix remodeling, transitioning to a reparative phase that begins about 7 days after MI and is marked by fibroblast activation, collagen deposition, and scar formation.

Cardiac remodeling following MI is a complex process involving inflammation, ECM dynamics, and cellular interactions (Figure 1). Understanding these mechanisms can aid in the development of targeted therapies to improve outcomes for patients with MI. Cardiac repair after MI is a dynamic, tightly regulated process involving the sequential and interdependent actions of multiple cell types. These interactions determine not only the immediate resolution of injury but also the long-term functional outcome for the myocardium. Continued research into the nuances of immune cell polarization, temporal regulation of ECM metabolism, and the translation of findings between animal models and patients remains critical. Bridging these knowledge gaps will be essential for the development of targeted therapies that optimize repair and prevent heart failure after MI.

## Figures and Tables

**Table 3:** Knowledge Gaps and Research Questions.

Identified Gap	Description	Proposed Research Questions
Neutrophil heterogeneity [18,118]	Temporal, spatial, and physiological diversity of neutrophil subpopulations	How do distinct neutrophil subsets regulate each phase of response?
Macrophage-fibroblast signaling [100]	Specific paracrine pathways for scar Optimization	What molecular signals determine the ECM traits of an optimal scar?
Antifibrotic therapy timing [1]	Risks of anti-inflammatory/pro-fibrotic intervention at various phases	When is the ideal window for anti-fibrotic modulation?
Regenerative approaches [119]	Limited cardiomyocyte regeneration after MI	How can cell therapy be guided by endogenous repair cues?
Sex differences [112,113,120,121]	Impact of sex on immune response and scar outcomes	What are the cellular/molecular bases for sex differences in MI response?

## Data Availability

Not applicable.

## Abbreviations

The following abbreviations are used in this manuscript:

Arg	Arginase
CD	Cluster differentiation
Csf	Colony-stimulating factor
Ccl	CC Chemokine ligand
Col	Collagen
Cxcl	CXC chemokine ligand
DAMPs	Damage-associated molecular patterns
ECM	Extracellular matrix
Fgf	Fibroblast growth factor
Fn	Fibronectin
Lgals	Galectin
Hmgb1	High-Mobility Group Box 1
Ifn	Interferon
IL	Interleukin
Ip	Interferon-gamma inducible protein
Lox	Lysyl oxidase
MCP	Monocyte chemotactic protein
M1	Pro-inflammatory
M2	Anti-inflammatory
MI	Myocardial infarction
MMP	Matrix metalloproteinase
Smad	Small mother against decapentaplegic
Sparc	Secreted protein acidic and rich in cysteine
Tgfβ	Transforming growth factor beta
TIMP	Tissue inhibitor of metalloproteinase
Tnfα	Tumor necrosis factor alpha
Tsp	Thrombospondin
Vegf	Vascular endothelial growth factor
